# Exploring Pain Researcher and Clinician Perceptions of Complementary, Alternative, and Integrative Medicine: A Large‐Scale, International Cross‐Sectional Survey

**DOI:** 10.1111/papr.70140

**Published:** 2026-03-14

**Authors:** Jeremy Y. Ng, Dennis Anheyer, Holger Cramer

**Affiliations:** ^1^ Institute of General Practice and Interprofessional Care University Hospital Tübingen Tübingen Germany; ^2^ Robert Bosch Center for Integrative Medicine and Health, Bosch Health Campus Stuttgart Germany; ^3^ Department of Health Research Methods, Evidence, and Impact, Faculty of Health Sciences McMaster University Hamilton Ontario Canada; ^4^ School of Public Health, Faculty of Health University of Technology Sydney Sydney New South Wales Australia; ^5^ Psychology and Psychotherapy; Methodology and Statistics in Psychology University of Witten/Herdecke Witten Germany

**Keywords:** alternative medicine, clinicians, complementary medicine, integrative medicine, pain, researchers

## Abstract

**Introduction:**

Complementary, alternative, and integrative medicine (CAIM) has gained popularity among patients experiencing pain, used alongside conventional medical treatments. This study aimed to explore the perceptions of pain clinicians and researchers on CAIM interventions.

**Methods:**

An anonymous, online survey was distributed to 46,223 authors who had published pain‐related research in MEDLINE‐indexed journals. The survey included multiple‐choice questions and open‐ended sections to gather detailed opinions.

**Results:**

A total of 1024 participants responded, 900 of which were eligible to participate; most identified as either pain researchers (*n* = 435/900, 48.33%) or both researchers and clinicians (*n* = 398/900, 44.22%). Many held senior positions (*n* = 549/892, 61.55%). Among the CAIM modalities, mind–body therapies such as meditation, yoga, and biofeedback were viewed as the most promising for pain prevention, treatment, and management, with 68.47% (*n* = 569/831) of participants endorsing these approaches. While (*n* = 341/777, 43.89%) of the respondents believed that most CAIM therapies are safe, only 25.55% (*n* = 198/775) disagreed with the idea that such therapies are effective. There was broad agreement on the need for more research into CAIM therapies, with 45.88% (*n* = 356/776) agreeing and 42.53% (*n* = 330/776) strongly agreeing that further investigation is valuable. Additionally, many respondents supported the inclusion of CAIM training in clinician education, either through formal programs (*n* = 361/778, 46.40%) or supplementary courses (*n* = 409/776, 52.71%).

**Conclusions:**

Mind–body therapies received the most positive feedback, while biofield therapies were met with the most skepticism. These findings highlight the interest in CAIM among pain researchers and clinicians and emphasize the need for more research and education tailored to this area.

**Abbreviations**
CAIM: complementary, alternative, and integrative medicine
IASP: International Association for the Study of Pain
MeSH: Medical Subject Headings
STROBE: STrengthening the Reporting of OBservational studies in Epidemiology

## 1 Introduction

Pain is one of the most common symptoms individuals experience worldwide. Among 146 countries worldwide, 34.1% of individuals were in pain in 2022 [1]. While it is common, the actual experience of pain is subject to great inter‐individual variability. Multiple biological and psychosocial variables contribute to these individual differences in pain, including demographic variables, genetic factors, and psychosocial processes. For example, sex, age, and ethnic group differences in the prevalence of pain conditions have been widely reported [2, 3, 4]. Recently, the International Association for the Study of Pain (IASP) updated its definition of pain to “an unpleasant sensory and emotional experience associated with, or resembling that associated with, actual or potential tissue damage” [5]. Based on chronicity, pain is commonly categorized as acute or chronic. Acute pain often presents due to a cause and has a shorter duration, whereas chronic pain is defined as pain that recurs or lasts longer than 3 months [6]. Whether acute or chronic, physicians often turn to a pharmaceutical standard of care when treating sensory or nociceptive pain, with the three most common being acetaminophen, non‐steroidal anti‐inflammatory drugs (NSAIDs), and opioids. All three of these classes are accompanied by a slew of side effects like nausea, hypertension, abdominal pain, and more [7]. As such, there is a need to evaluate cost‐effective and lower‐risk nonpharmacological treatments. This creates a need for patients and clinicians to look toward other forms of healthcare to alleviate their pain and discomfort [8].

Complementary, alternative, and integrative medicine (CAIM) has gained increasing popularity over the past decades as a potential treatment option among patients [8]. According to the US National Center for Complementary and Integrative Health (NCCIH), “complementary medicine” is defined as a non‐mainstream approach that is used in conjunction with conventional medicine, whereas “alternative medicine” is when the non‐mainstream approach replaces the traditional approach [9, 10]. Integrative health emphasizes a multimodal approach wherein multiple conventional health approaches are utilized alongside complementary approaches [9]. Some common CAIM therapies that are currently used for the management of pain include acupuncture, chiropractic manipulation, massage, and meditation [11]. For example, acupuncture is a practice originating from the East Asian subcontinent that is often practiced to alleviate pain. Despite over 80 systematic reviews being conducted so far to evaluate the impact of acupuncture on pain relief, these reviews have certain degrees of conflict between them, indicating that further research is needed [12]. Chiropractic manipulation is a treatment often sought out by patients experiencing various types of joint‐related pain. Chiropractors strongly rely on the use of manual therapy, particularly spinal manipulation, which is the main form of care they provide [13]. These therapies are often used in conjunction with conventional therapies like pharmaceutical medications and surgery, or as a stand‐alone. However, despite the growing popularity of CAIM in pain management, there are unique barriers to the conduct and application of CAIM research [14]. With regards to pain specifically, clinical practice guidelines for a range of CAIMs for the management of pain are uncertain overall, suggesting a need for the generation of more and higher‐quality evidence on CAIMs [15, 16, 17, 18]. This presents a clear challenge to clinicians and researchers who are interested in exploring the potential benefits of these therapies. While some clinicians view CAIM as a valuable addition to conventional healthcare, others remain skeptical of its effectiveness and safety [19]. Thus, there is a clear divide in opinion which has led to varied approaches when integrating CAIM into clinical settings. To understand their perceptions better, we conducted a large‐scale, cross‐sectional, international study to gather data from pain researchers and clinicians across various geographic regions on their perceptions about CAIM.

## 2 Methods

### 2.1 Transparency Statement

We sought approval and were granted clearance from the University Hospital Tubingen Research Ethics Board to conduct this study (REB Number: 389/2023BO2). Our protocol was registered on the Open Science Framework (OSF) prior to commencing participant recruitment [20]. All our study materials and data are also available on OSF: https://doi.org/10.17605/OSF.IO/DVECG.

### 2.2 Study Design

We conducted an anonymous, online, cross‐sectional survey consisting of corresponding authors who have published articles about or relating to pain in journals indexed in MEDLINE [21].

### 2.3 Sampling Framework

A search of the Ovid MEDLINE(R) ALL database was conducted using a series of pain‐related Medical Subject Headings (MeSH) to identify relevant publications. The search was limited to English‐language records published between May 1, 2016, and May 1, 2023, yielding a complete set of 121,155 PubMed Identifiers (PMIDs). The complete search strategy is provided in Table 1; the selection of MeSH terms was comprehensively reviewed by JYN, who has over 10 years of experience in bibliographic database search strategy development. These PMIDs were exported as a CSV file and processed in R using a custom script built with the easyPubMed package to extract the corresponding authors' names, institutional affiliations, and email addresses for inclusion in this study [22].

**TABLE 1:** Strategy for author name and email address retrieval.

Database: Ovid MEDLINE(R) ALL < 1946 to May 23, 2023 > search strategy
1. exp. Pain, Referred/ or exp. Pleasure‐Pain Principle/ or exp. Pain, Procedural/ or exp. Chronic Pain/ or exp. Shoulder Pain/ or exp. Acute Pain/ or exp. Pain Management/ or exp. Abdominal Pain/ or exp. Pain Measurement/ or exp. Nociceptive Pain/ or exp. Pain, Intractable/ or exp. Visceral Pain/ or exp. Pelvic Pain/ or exp. Eye Pain/ or exp. Pain, Postoperative/ or exp. Neck Pain/ or exp. Pelvic Girdle Pain/ or exp. Labor Pain/ or exp. Pain Insensitivity, Congenital/ or exp. Low Back Pain/ or exp. Pain Threshold/ or exp. Pain/ or exp. Patellofemoral Pain Syndrome/ or exp. Myofascial Pain Syndromes/ or exp. Flank Pain/ or exp. Chest Pain/ or exp. Back Pain/ or exp. Facial Pain/ or exp. Musculoskeletal Pain/ or exp. Pain Clinics/ or exp. Complex Regional Pain Syndromes/ or exp. Breakthrough Pain/ or exp. Pain Perception/ or exp. Cancer Pain/ (511661)
2. limit 1 to english language (434060)
3. limit 2 to dt = 20,160,501–20,230,501 (121155)

### 2.4 Participant Recruitment

The authors who were identified by utilizing our sampling framework were contacted and asked to complete the survey. Initially, the list of emails went through a screening process where duplicate and/or invalid emails were removed from the dataset. Any names that were extracted incorrectly were corrected; any names that did not have an email associated with them were also removed. The software SurveyMonkey was utilized to send emails to all the authors [23]. This email included an explanation of the study, the study's goals, as well as a link to the survey. Upon clicking the link, participants were initially taken to an informed consent form. The survey began once participants consented to the terms and conditions outlined and indicated their intention to participate. After the initial email was sent on September 04, 2023, subsequent reminders were sent on September 18, 2023, October 04, 2023, and October 18, 2023. After the final reminder was sent, the survey was left open for 4 weeks. Participants could skip any questions at their discretion; no financial compensation was provided for completing the survey.

### 2.5 Survey Design

The initial part of the survey consisted of a screening question asking the participant if they were a researcher or clinician within the field of pain, followed by a series of demographic questions to establish certain characteristics of the participant. Patients and the general public were not eligible to partake in the survey. The second part of the survey asked the respondents about their perceptions of CAIM. The survey consisted of 30 multiple choice questions and one open‐ended question at the end. To our knowledge, no existing survey instrument adequately addressed the specific objectives of this study; therefore, a new survey was developed to explore researchers' and clinicians' perceptions of CAIM within the field of pain. Prior to distribution, the survey was pilot‐tested independently by two experienced CAIM researchers to assess its clarity, flow, and relevance. Their feedback led to minor revisions in the wording of several questions to improve interpretability and ensure that the items were appropriately targeted to the intended audience. This process helped enhance the face validity and overall usability of the final survey instrument. The complete copy of the survey is provided on OSF: https://osf.io/r4cfh.

### 2.6 Data Management and Analysis

The survey data was analyzed and basic descriptive statistics, such as counts and percentages, were computed. To analyze the qualitative data, a coding process was performed where similar groups of ideas were assigned a code, which was a concise interpretation of the response provided. Thematic analysis was conducted to narrow down the codes and group similar ones together. The results were reported using the STrengthening the Reporting of OBservational studies in Epidemiology (STROBE) checklist [24, 25].

## 3 Results

### 3.1 Demographics

Using the aforementioned search strategy, a total of 121,155 records were retrieved from which 46,223 unique corresponding author names and email addresses were identified through data extraction in R using the easyPubMed package followed by data cleaning. Of the 46,223 emails sent out, 25,316 emails were unopened, 14,477 were opened and 6430 bounced. A total of 1024 responses were received, thus the response rate for all emails was 2.57% for received emails (i.e., opened and unopened emails), and 7.07% for opened emails. It is important to acknowledge that not every participant answered all questions, therefore, we present the total number of responses for each question as fractions. Responses were considered incomplete if no answers were provided beyond the initial screening question. The survey took an average of 9 minutes and 47 second to complete. Most respondents identified as only pain researchers (*n* = 435/900, 48.33%) or both a pain researcher and clinician (*n* = 398/900, 44.22%). Far fewer respondents identified as only a pain clinician (*n* = 67/900, 7.44%). Finally, some responded as neither a researcher nor a clinician (*n* = 98); these individuals are not part of the 900 viable responses received, and such participants were excluded from partaking in the remainder of the survey. Respondents were almost equally split between male (*n* = 431/893, 48.26%) and female (*n* = 443/893, 49.61%), and most were between the ages of 35–44 (*n* = 267/895, 29.83%) or 45–54 (*n* = 257/895, 28.72%). Most participants indicated that they were not part of a visible minority group (*n* = 728/894, 81.43%). With respect to WHO World Regions, participants were primarily located in Europe (*n* = 349/891, 39.17%) or the Americas (*n* = 318/891, 35.69%). Respondents also primarily identified as a faculty member/principal investigator (*n* = 448/894, 50.11%) or a clinician (*n* = 374/894, 41.83%) in a senior career stage (*n* = 549/892, 61.55%). When asked to describe their primary research area, most of the respondents stated clinical research (*n* = 571/777, 73.49%). The characteristics of participants can be found in Table 2. The complete raw, deidentified survey data is provided on OSF: https://osf.io/s3jtu. Crosstabs for key demographic variables can also be found on OSF: https://osf.io/dvecg.

**TABLE 2:** Characteristics of survey participants.

Self‐identification as researcher or clinician (*n* = 900)	
Researcher within field of pain only	435 (48.33%)
Clinician within field of pain only	67 (7.44%)
Both researcher and clinician within field of and pain	398 (44.22%)
Sex (*n* = 893)	
Male	431 (48.26%)
Female	443 (49.61%)
Prefer not to say	13 (1.46%)
Prefer not to self‐describe	6 (0.67%)
Age (*n* = 895)	
Under 18	1 (0.11%)
18–24	1 (0.11%)
25–34	78 (8.72%)
35–44	267 (29.83%)
45–54	257 (28.72%)
55–64	191 (21.34%)
> 65	91 (10.17%)
Prefer not to say	9 (1.01%)
Visible minority (*n* = 894)	
Yes	138 (15.44%)
No	728 (81.42%)
Prefer not to say	28 (3.13%)
Location (*n* = 891)	
Africa	22 (2.47%)
Americas	318 (35.69%)
Eastern Mediterranean	40 (4.49%)
Europe	349 (39.17%)
South‐East Asia	78 (8.75%)
Western Pacific	75 (8.42%)
Prefer not to say	9 (1.01%)
Current position (*n* = 894)	
Clinician student	2 (0.22%)
Clinician	374 (41.83%)
Graduate student	30 (3.36%)
Postdoctoral fellow	76 (8.50%)
Faculty member/Principal investigator	448 (50.11%)
Research support staff (e.g., research manager, research associate, technician)	38 (4.25%)
Scientist in academia	266 (29.75%)
Scientist in industry	18 (2.01%)
Scientist in third sector	11 (1.23%)
Government scientist	19 (2.13%)
Other (please specify)	38 (4.25%)
Career stage (*n* = 892)	
Graduate or clinician student	15 (1.68%)
Early career researcher (< 5 years post education)	121 (13.57%)
Mid‐career research (5–10 years post education)	207 (23.21%)
Senior researcher (> 10 years post education)	549 (61.55%)
Primary research area (*n* = 777)	
Clinical research	571 (73.49%)
Preclinical research (in vivo)	135 (17.37%)
Preclinical research (in vitro)	65 (8.37%)
Health systems research	76 (9.78%)
Health services research	160 (20.59%)
Methods research	100 (12.87%)
Epidemiological research	129 (16.60%)
Other	35 (4.50%)
Area of CAIM Research (*n* = 776)	
Mind–body therapies	191 (24.61%)
Biologically based practices	139 (17.91%)
Manipulative and body‐based practices	151 (19.46%)
Biofield therapy	18 (2.32%)
Whole medical systems	99 (12.76%)
I have never conducted any CAIM research	324 (41.75%)
Other	62 (7.99%)

Abbreviation: CAIM, complementary, alternative, and integrative medicine.

### 3.2 CAIM Practices and Experiences

When asked about conducting CAIM research, while some respondents had never conducted CAIM research (*n* = 324/776, 41.75%), of those who had, mind–body therapies were the most common research area (*n* = 191/776, 24.61%). As shown in Table 3, participants perceived that mind–body therapies to be the most promising with regards to the prevention, treatment, or management of conditions associated with pain (*n* = 569/831, 68.47%) along with manipulative and body‐based practices (*n* = 336/831, 40.43%). A few participants reported that they did not perceive any CAIM categories to be promising (*n* = 82/831, 9.87%).

**TABLE 3:** Perspectives on most promising CAIM categories.

**(*n* = 831)**	
Mind body therapies	569 (68.47%)
Biologically based practices	241 (29.00%)
Manipulative and body‐based practices	336 (40.43%)
Biofield therapies	63 (7.58%)
Whole medical systems	271 (32.61%)
I do not perceive any CAIM categories to be promising	82 (9.87%)
Other	52 (6.26%)

Abbreviation: CAIM, complementary, alternative, and integrative medicine.

As described in Table 4, the CAIM categories for which patients had most often disclosed use were manipulative and body‐based therapies (*n* = 320/418, 76.56%), mind–body therapies (*n* = 297/418, 71.05%), biologically based practices (*n* = 277/418, 66.27%), or whole medical systems (*n* = 273/418, 65.31%). When asked about the percentage of their patients who have disclosed or sought to use CAIM therapies, the majority of respondents stated either 11%–20% (*n* = 86/411, 20.92%), 0%–10% (*n* = 84/411, 20.44%), or 21%–30% (*n* = 71/411, 17.27%). Over two thirds of clinicians had practiced or recommended mind–body therapies to their patients (*n* = 267/415, 64.34%), and half of physicians had recommended manipulative and body‐based practices to their patients before (*n* = 194/415, 46.75%).

**TABLE 4:** Patient preferences.

CAIM interventions patients have sought counseling or disclosed using: (*n* = 418)	
Mind body therapies	297 (71.05%)
Biologically based practices	277 (66.27%)
Manipulative and body‐based practices	320 (76.56%)
Biofield therapies	124 (29.67%)
Whole medical systems	273 (65.31%)
I have never had a patient seek counseling or disclose using CAIM	29 (6.94%)
Percentage of patients who have disclosed using CAIM or seek counseling on CAIM therapies: (*n* = 411)	
0%–10%	84 (20.44%)
11%–20%	86 (20.92%)
21%–30%	71 (17.27%)
31%–40%	42 (10.22%)
41%–50%	32 (7.79%)
51%–60%	26 (6.33%)
61%–70%	15 (3.65%)
71%–80%	19 (4.62%)
81%–90%	10 (2.43%)
91%–100%	26 (6.33%)
Areas that participants have practiced or recommended CAIM to patients: (*n* = 415)	
Mind body therapies	267 (64.34%)
Biologically based practices	136 (32.77%)
Manipulative and body‐based practices	194 (46.75%)
Biofield therapies	25 (6.02%)
Whole medical systems	125 (30.12%)
I have never practiced nor recommended CAIM to my patients	60 (14.46%)
Other	19 (4.58%)

Abbreviation: CAIM, complementary, alternative, and integrative medicine.

### 3.3 CAIM Training and Implementation

As shown in Table 5, nearly half of the respondents reported not having any formal training in CAIM specialties (*n* = 199/415, 47.95%). Out of the respondents who received formal training, the top two common fields were manipulative and body‐based practices (*n* = 114/415, 27.47%) and mind–body therapies (*n* = 104/415, 25.06%). With regards to supplemental training, the responses were varied among categories, with most participants having received training in mind–body therapy (*n* = 202/413, 48.91%), manipulative and body‐based practices (*n* = 124/413, 30.02%), biologically based practices (*n* = 120/413, 29.06%), and whole medical systems (*n* = 105/413, 25.42%). A similar number of individuals had not received any supplemental training (*n* = 122/413, 29.54%). Over half of the respondents reported being asked about CAIM occasionally (*n* = 431/828, 52.05%), while nearly a third of the respondents had been asked about CAIM often (*n* = 269/828, 32.49%). The remaining respondents reported having never been asked about CAIM (*n* = 128/828, 15.46%). When asked about where participants would seek out information regarding CAIM, the answer was overwhelmingly through academic literature (*n* = 765/831, 92.06%), with the next option being through conference presentations or workshops (*n* = 438/831, 52.71%). Colleagues (*n* = 293/831, 35.26%) and continuing education courses (*n* = 279/831, 33.57%) were also among sources that participants would likely seek information from (Table 6).

**TABLE 5:** Participant training in CAIM areas.

Formal training (*n* = 415)	
Mind body therapies	104 (25.06%)
Biologically based practices	56 (13.49%)
Manipulative and body‐based practices	114 (27.47%)
Biofield therapies	12 (2.89%)
Whole medical systems	53 (12.77%)
I have not received formal training in any of these areas	199 (47.95%)
Other	17 (4.10%)
Supplementary training (*n* = 413)	
Mind body therapies	202 (48.91%)
Biologically based practices	120 (29.06%)
Manipulative and body‐based practices	124 (30.02%)
Biofield therapies	26 (6.30%)
Whole medical systems	105 (25.42%)
I have not received supplemental training in any of these areas	122 (29.54%)
Other	11 (2.66%)

Abbreviation: CAIM, complementary, alternative, and integrative medicine.

**TABLE 6:** Participant knowledge related to CAIM.

Members outside of research/clinic asking about CAIM (*n* = 828)	
Yes, I have been asked about CAIM often	269 (32.49%)
Yes, I have been asked about CAIM occasionally	431 (52.05%)
No, I have never been asked about CAIM	128 (15.46%)
Where to seek out CAIM information (*n* = 831)	
Academic literature	765 (92.06%)
Colleagues	293 (35.26%)
Conference presentations or workshops	438 (52.71%)
Continuing education course	279 (33.57%)
Formal clinical training	160 (19.25%)
Government agencies	142 (17.09%)
Health or information pages on the internet	233 (28.04%)
Local or national news	27 (3.25%)
Personal experience	133 (16.00%)
Social media	41 (4.93%)
Working with patients/clients	171 (20.58%)
Other (please specify)	17 (2.05%)

Abbreviation: CAIM, complementary, alternative, and integrative medicine.

### 3.4 General CAIM Perceptions

**FIGURE 1:**
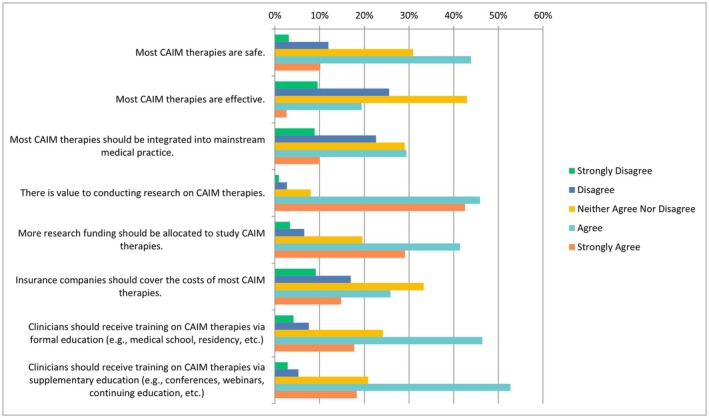
Agreement regarding CAIM therapies in general. CAIM, complementary, alternative, and integrative medicine.

In the following questions, respondents were provided with a scale including the following options: “strongly disagree”, “disagree”, “neither agree or disagree”, “agree” or “strongly agree”. When asked about CAIM therapies in general, nearly half of participants selected “agree” to the statement “there is value to conducting research on CAIM therapies” (*n* = 356/776, 45.88%) with a similar number of participants who strongly agreed (*n* = 330/776, 42.53%), as shown in Figure 1. Similarly, when asked about their level of agreement with the statement “clinicians should receive training on CAIM therapies via formal education”, most respondents ranked this statement as “agree” (*n* = 361/778, 46.40%) or “strongly agree” (*n* = 138/778, 17.74%). Most of the respondents selected “agree” when asked whether clinicians should receive training via supplementary education as well (*n* = 409/776, 52.71%). Respondents were split when asked whether they would be comfortable counseling their patients about most CAIM therapies. Roughly a quarter of patients selected “disagree” (*n* = 100/391, 25.58%) and another quarter for “neither agree nor disagree” (*n* = 101/391, 25.83%) while a third of patients agreed (*n* = 127/391, 32.48%). In terms of recommending CAIM to their patients, perceptions were mixed, whereby the majority of the responses were split between “disagree”, “neither agree or disagree”, or “agree”.

### 3.5 Specific CAIM Perceptions

**FIGURE 2:**
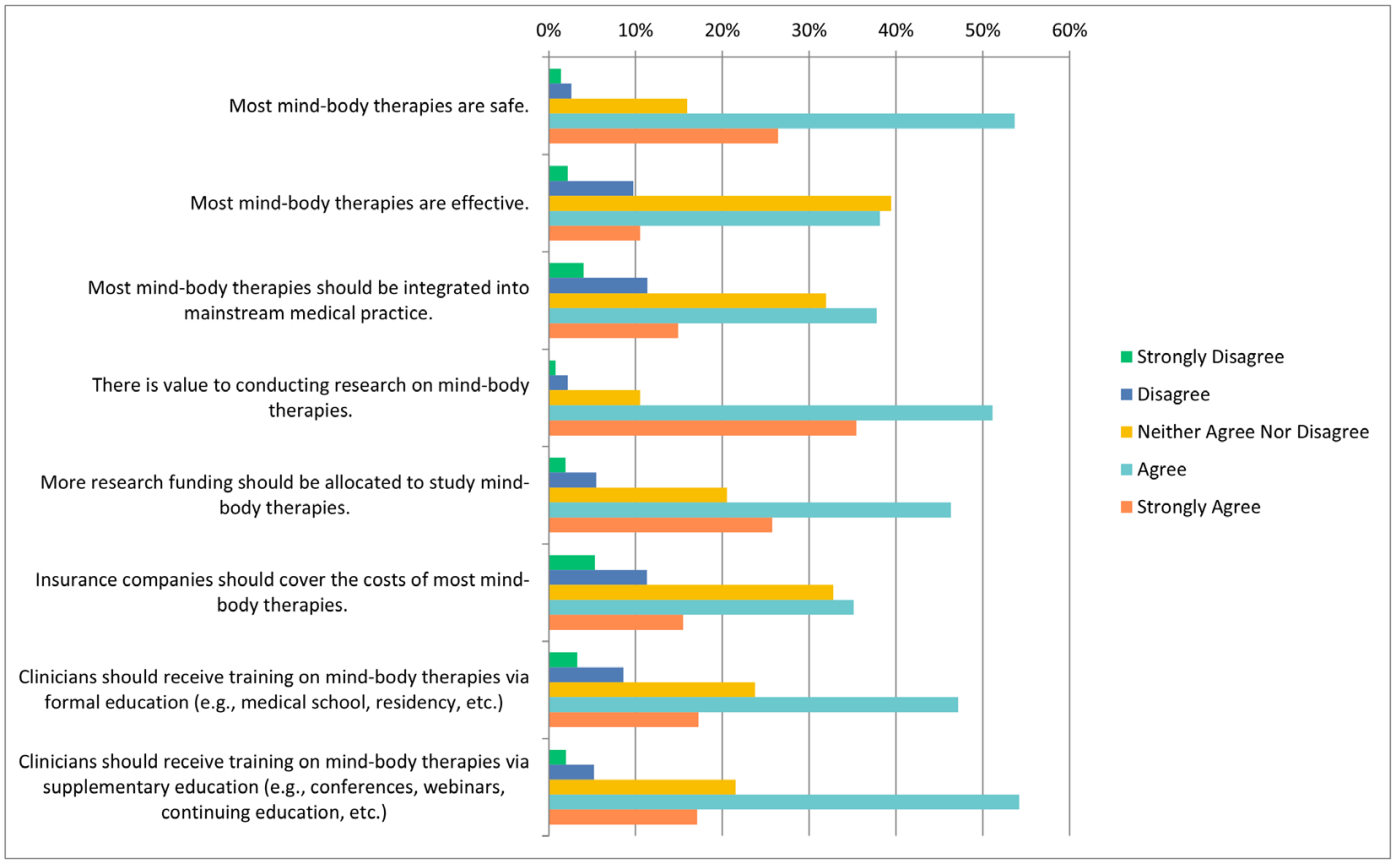
Agreement regarding statements about mind–body therapies.

**FIGURE 3:**
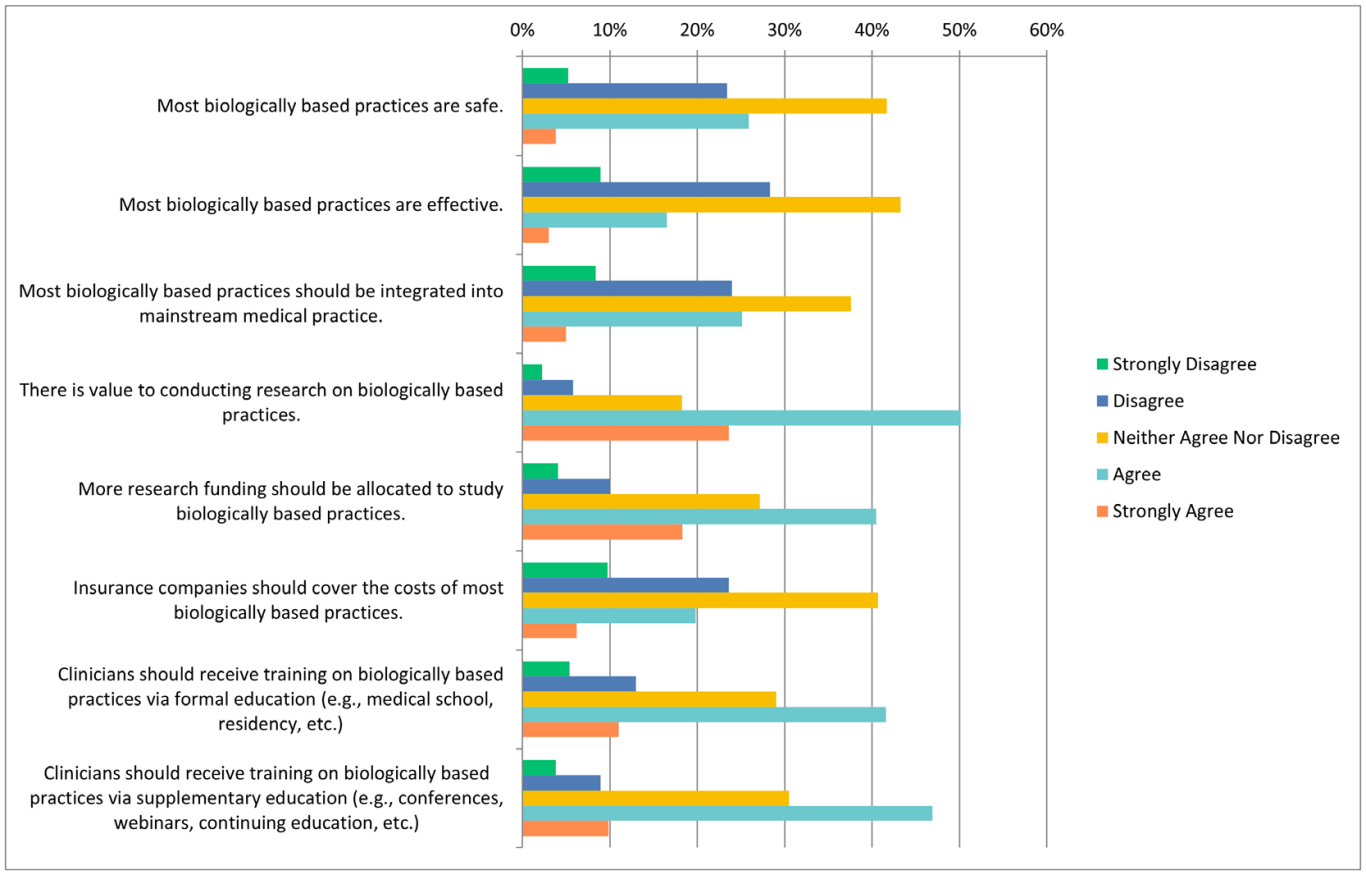
Agreement regarding statements about biologically based practices.

**FIGURE 4:**
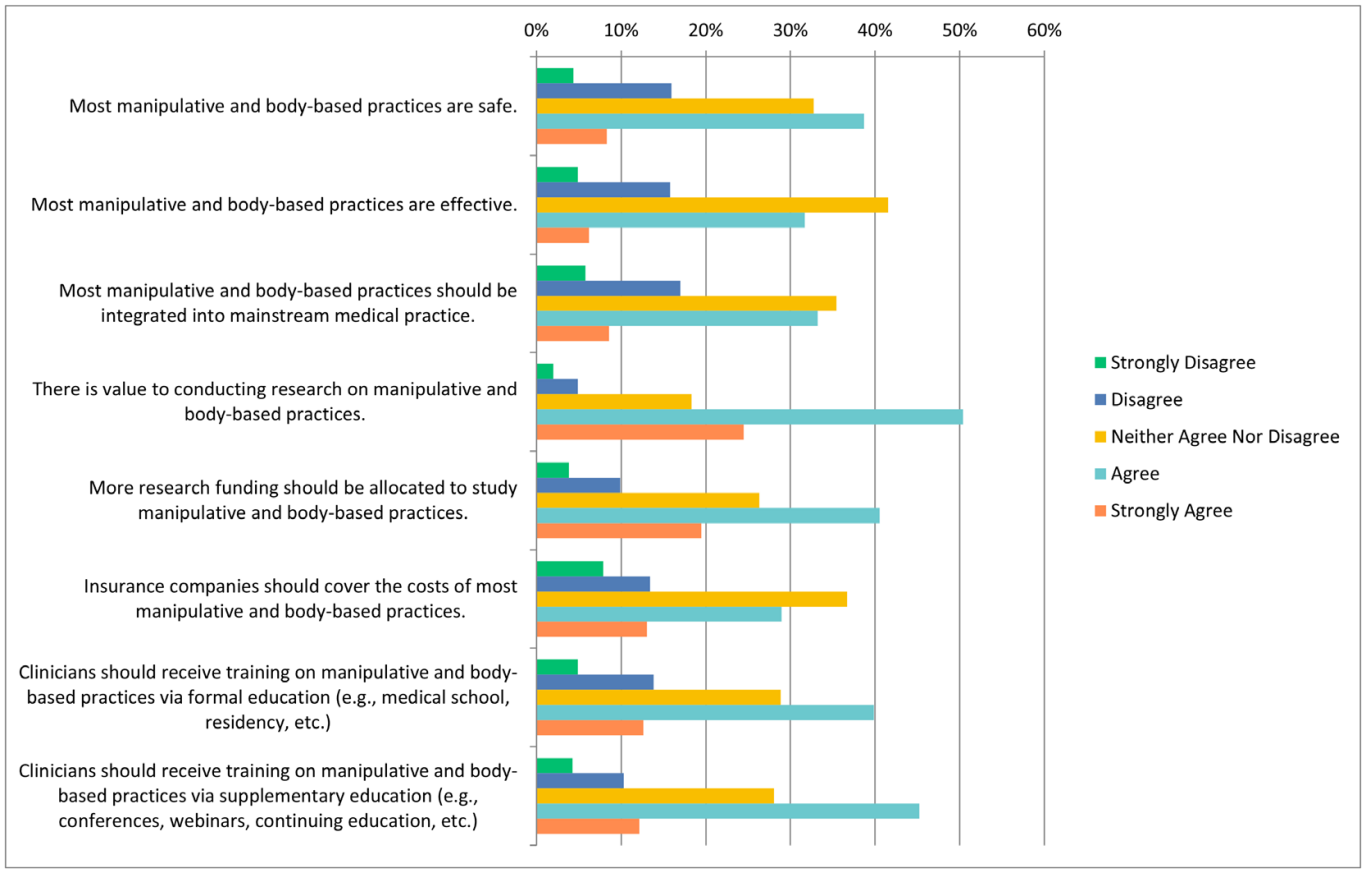
Agreement regarding statements about manipulative and body‐based practices.

**FIGURE 5:**
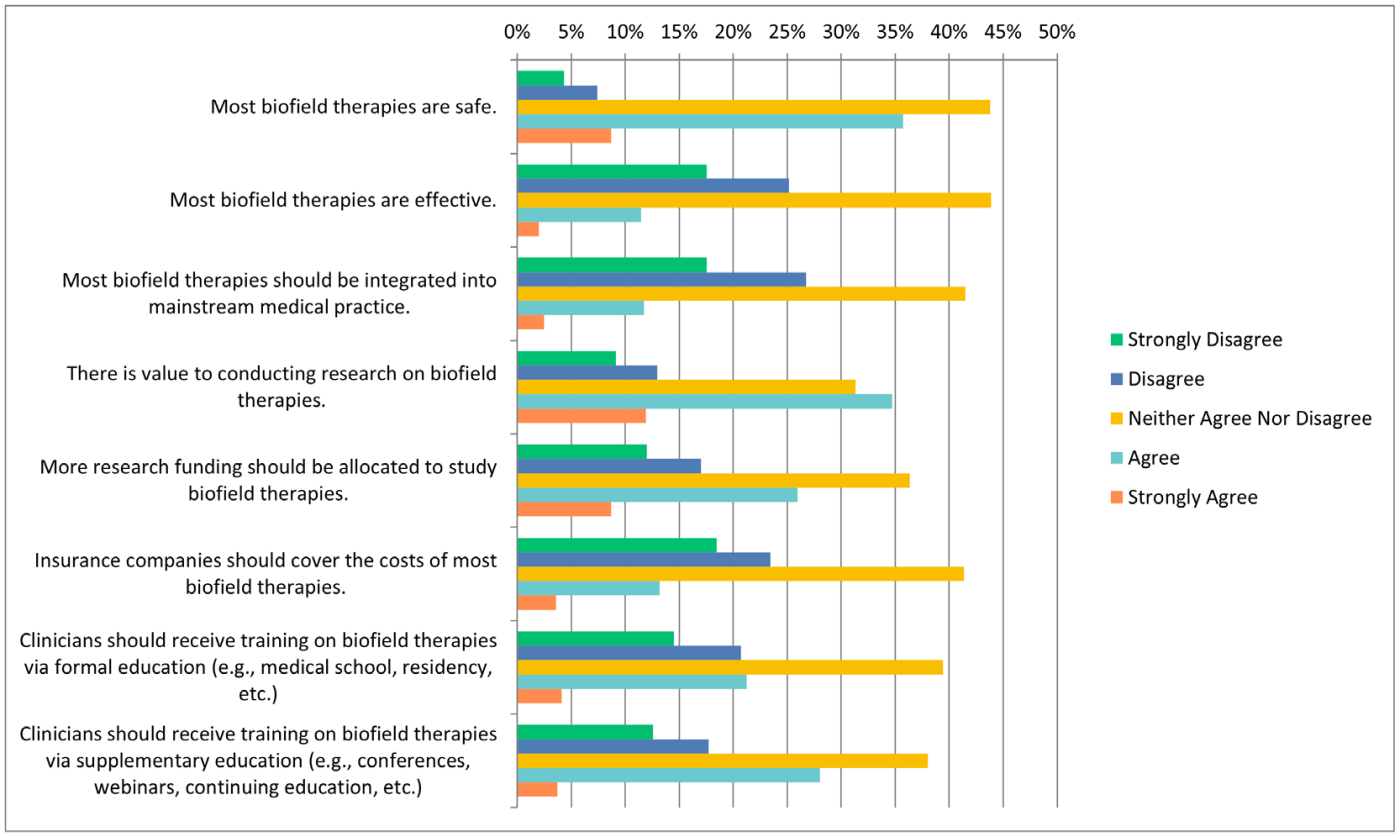
Agreement regarding statements about biofield practices.

**FIGURE 6:**
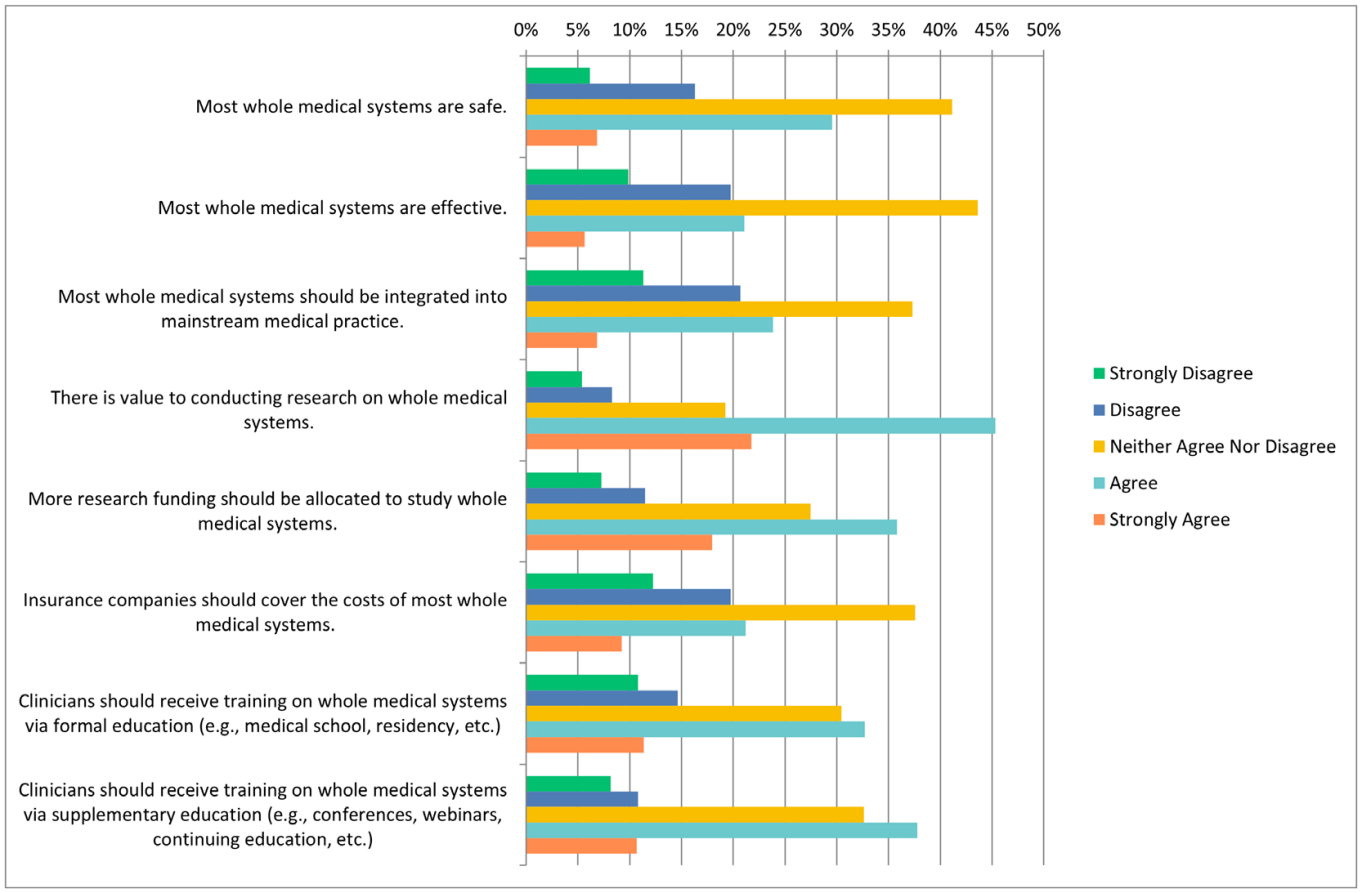
Agreement regarding statements about whole medical systems.

The following questions had the same ranking scheme as the previous question set, with references to each specific CAIM category including mind–body therapies, biologically based practices, manipulative and body‐based practices, biofield therapies, and whole medical systems (Figures 2, 3, 4, 5, 6).

#### 3.5.1 Perceptions of Mind–Body Therapy

With regards to mind–body therapies (e.g., meditation, biofeedback, hypnosis, yoga, tai chi, imagery, creative outlets), over half of the respondents selected “agree” that most mind–body therapies are safe (*n* = 414/772, 53.63%) with more than a quarter selecting “strongly agree” (*n* = 204/772, 26.42%). A total of 37.77% (*n* = 292/773) individuals chose “agree” for the statement that most mind–body therapies should be integrated into mainstream medical practice. The majority of respondents either “agreed” (*n* = 394/771, 51.10%) or “strongly agreed” (*n* = 273/771, 35.41%) that there is value to conducting research on mind–body therapies. Almost half of the respondents (*n* = 356/769, 46.29%) selected “agree” that more research funding should be allocating to studying mind–body therapies. When asked about receiving mind–body training during clinician training, 47.13% of respondents (*n* = 361/766) selected “agree” that training should be received via formal education whereas 54.18% of respondents (*n* = 415/766) selected “agree” that it should be received via supplementary education (Figure 2). When asked about counseling patients about most mind–body therapies to patients, 42.71% of (*n* = 167/391) respondents selected “agree”, and 13.04% selected “disagree” (*n* = 51/391). In terms of recommending mind–body therapies to patients, 40.36% (*n* = 157/389) of respondents selected “agree”, 21.85% (*n* = 85/389) selected “neither agree nor disagree”, and 16.45% (*n* = 64/389) selected “disagree”.

#### 3.5.2 Perceptions of Biologically Based Practices

41.70% of participants (319/765) selected “neither agree nor disagree” on the safety of these practices, while 43.25% (*n* = 330/763) chose “agree” that most biologically based practices are effective. Regarding integration into mainstream medical practice, the opinions were more varied as 37.57% (*n* = 287/764) selected “neither agree nor disagree” and 25.13% (*n* = 192/764) selected “agree”. The majority (*n* = 382/762, 50.13%) selected “agree” that there is value in conducting research on biologically based practices, and a large proportion (*n* = 307/759, 40.45%) selected “agree” that more research funding should be allocated to studying them. However, opinions on insurance coverage were mixed, as 40.68% (*n* = 310/762) selected “neither agree nor disagree”, while 23.62% (*n* = 180/762) chose “disagree”. With regards to clinician training, participants selected “agree” in regards to receiving both formal (*n* = 317/762, 41.60%) and supplementary education (*n* = 357/761, 46.91%) (Figure 3). When asked about counseling patients about biologically based therapies, 29.46% of respondents (*n* = 114/387) selected “agree”, while less than a quarter of respondents selected “disagree” (*n* = 96/387, 24.81%). Around a third of respondents chose “disagree” regarding recommending biologically based practices (*n* = 125/387, 32.30%), whereas a fifth selected “agree” with regards to recommending such practices (*n* = 79/387, 20.41%). Additionally, 11.63% (*n* = 45/387) selected “strongly disagree” to counseling, and 13.95% (*n* = 54/387) selected “strongly disagree” for recommending biologically based practices.

#### 3.5.3 Perceptions of Manipulative and Body‐Based Practices

38.68% of participants (294/760) selected “strongly agree” that most manipulative and body‐based practices are safe, while a proportion of 31.67% (*n* = 241/761) chose “agree” that these practices are effective. Concerning integration into mainstream medical practice, respondents were divided, with 35.44% (*n* = 269/759) selecting “neither agree nor disagree” and 33.20% (*n* = 252/759) selecting “disagree”. Additionally, half of the respondents (*n* = 383/760, 50.39%) selected “agree” that there was value in conducting research about such practices. A notable number (*n* = 308/760, 40.53%) selected “agree” to the statement that more research funding should be allocated to studying these practices. Opinions on insurance coverage varied, with 36.71% (*n* = 279/760) selecting “neither agree nor disagree” that insurance companies should cover the costs, while 28.95% (*n* = 220/760) selected “agree” and 13.42% (*n* = 102/760) selected “disagree”. Regarding clinician training, more than a third of respondents (*n* = 303/760, 39.87%) selected “agree” that clinicians should receive training through medical school or residency, with another 12.63% (*n* = 96/760) choosing “strongly agree”. With regards to supplementary education, 45.24% (*n* = 342/756) of participants selected “agree” and 12.17% (*n* = 92/756) selected “strongly agree” (Figure 4). When asked about whether respondents would counsel their patients regarding manipulative and body‐based practices, 14.55% selected “strongly agree” (*n* = 56/385), 35.32% selected “agree” (*n* = 136/385), 24.42% selected “neither agree nor disagreed” (*n* = 94/385), 19.22% selected “disagree” (*n* = 74/385) and 6.49% selected “strongly disagree” (*n* = 25/385). The results were more divided when asked about recommending biologically based practices to their patients. A quarter of individuals chose “disagree” (*n* = 98/385, 25.45%), another quarter of individuals selected “neither agree nor disagree” (*n* = 95/385, 24.68%) and 28.05% (*n* = 108/385) of individuals selected “agree”.

#### 3.5.4 Perceptions of Biofield Therapies

More than a third of participants (*n* = 271/758, 35.75%) selected “agree” to the statement that most biofield therapies are safe, while a large proportion of participants (*n* = 332/758, 43.80%) selected “neither agree nor disagree”. Opinions with regards to the effectiveness of biofield therapies were more negatively perceived, with a quarter of participants selecting “disagree” (*n* = 191/759, 25.16%) and 17.52% of individuals selecting “strongly disagree” (*n* = 133/759). When asked about the integration of biofield therapies into mainstream practices, 17.52% of respondents selected “strongly disagree” (*n* = 133/759), over a quarter of respondents selected “disagree” (*n* = 203/759, 26.75%), and less than half of individuals selected “neither agree nor disagree” (*n* = 315/759, 41.50%). Regarding the value of research, 34.74% (*n* = 263/757) selected “agree” that there is value in conducting research on biofield therapies, while 31.31% (*n* = 237/757) chose “neither agree nor disagree”. Additionally, a quarter of participants (*n* = 197/759, 25.96%) selected “agree” that more research funding should be allocated to study biofield therapies. In terms of insurance coverage, opinions varied, with 23.45% of individuals selecting “disagree” that insurance companies should cover the costs of most biofield therapies (*n* = 178/759), and 41.37% selected “neither agree nor disagree” (*n* = 314/759). Regarding clinician training, there were similar results for both formal and supplementary education (Figure 5). The majority of participants either selected “strongly disagree” (*n* = 76/383, 19.84%) or “disagree” (*n* = 119/383, 31.07%) with the statement that they would be comfortable counseling patients about most biofield therapies. Similarly, for recommending these therapies, over half of participants selected either “strongly disagree” (*n* = 102/383, 26.63%) or “disagreed” (*n* = 125/383, 32.64%).

#### 3.5.5 Perceptions of Whole Medical Systems

Notably, 41.13% (*n* = 313/761) of participants selected “neither agree nor disagree” with respect to the safety of most whole medical systems, while a similar percentage (*n* = 331/759, 43.61%) selected “neither agree nor disagree” regarding their effectiveness. Regarding integration into mainstream medical practice, most respondents (*n* = 283/759, 37.29%) selected “neither agreed nor disagreed”. Participants acknowledged the value of research on whole medical systems, with 45.32% (*n* = 344/759) choosing “agree”, and 35.80% (*n* = 271/757) of respondents selecting “agree” regarding providing more research funding. Regarding insurance coverage, 37.55% (*n* = 285/759) selected “neither agree nor disagree”. In terms of clinician training, 32.72% (*n* = 248/758) selected “agree” that formal education should include whole medical systems, while 37.76% (*n* = 287/760) chose “agree” that it training should be provided via supplementary education (Figure 6). For counseling, 28.24% (*n* = 109/386) selected “neither agree nor disagree”, while 24.35% (*n* = 94/386) selected “agree”. In contrast, 15.28% (*n* = 59/386) selected “strongly disagree” with counseling. Regarding recommending these therapies to patients, most participants selected either “neither agree nor disagree” (*n* = 109/385, 28.31%) or “disagree” (*n* = 103/395, 26.75%). A smaller percentage, 6.49% (*n* = 25/385), strongly agreed with recommending whole medical systems to patients.

### 3.6 Perceived Benefits and Challenges

When asked about the benefits associated with CAIM, of 748 participants, 74.33% (*n* = 556) stated “expanded treatment options for patients”. The second and third most voted options were “holistic approach to wellness” (*n* = 507, 67.78%) and “empowerment of patients to take control of their own health” (*n* = 477, 63.77%) respectively. Of the 46 respondents (*n* = 46, 6.15%), who provided their own response under “other”, the common themes that emerged were (1) there are no benefits of CAIM, (2) CAIM is effective in reducing pain, and (3) reduced medication use (Table 7). Additionally, participants were asked about challenges that they perceive are associated with CAIM. Out of 755 participants, 87.5% (*n* = 661) believed that the lack of scientific evidence for safety and efficacy was the biggest challenge. Following closely behind as the highest voted challenge was lack of standardization in product quality and dosing (*n* = 627, 83.05%) and limited regulation and oversight (*n* = 522, 69.14%). When asked to further share their opinions via an “other option”, 5.70% participants (*n* = 43) provided further thoughts with varying themes. Some of the common themes that emerged were misrepresentation of effectiveness, lack of standardization, and risk of false hope (Table 7).

**TABLE 7:** Benefits and challenges associated with CAIM.

Benefits (*n* = 748)	
Expanded treatment options for patients	556 (74.33%)
Holistic approach to health and wellness	507 (67.78%)
Empowerment of patients to take control of their own health	477 (63.77%)
Fewer side effects than conventional medicine	326 (43.58%)
Increased patient satisfaction and well‐being	391 (52.27%)
Focus on prevention and lifestyle changes	459 (61.36%)
Cultural and spiritual relevance for certain populations	379 (50.67%)
Potential cost savings for patients and healthcare systems	278 (37.17%)
Potential to address chronic health conditions that conventional medicine has been unable to treat effectively	403 (53.88%)
Integration with conventional medicine to provide CAIM options	361 (48.26%)
Other (please specify)	46 (6.15%)
Challenges (*n* = 755)	
Lack of scientific evidence for safety and efficacy	661 (87.55%)
Lack of standardization in product quality and dosing	627 (83.05%)
Limited regulation and oversight	522 (69.14%)
Limited integration with mainstream healthcare systems	430 (56.95%)
Stigmatization and skepticism from healthcare providers and the public	346 (45.83%)
Limited availability in certain geographic areas or for certain populations	298 (39.47%)
High cost and lack of insurance coverage	370 (49.01%)
Potential interactions with prescription medicine	325 (43.05%)
Limited patient education and understanding	352 (46.62%)
Difficulty in distinguishing legitimate practices from scams or fraudulent claims	532 (70.46%)
Other (please specify)	43 (5.70%)

Abbreviation: CAIM, complementary, alternative, and integrative medicine.

### 3.7 Thematic Analysis

When respondents were asked if they had anything else to share about their perceptions of CAIM, 797 individuals skipped the question, however, 236 individuals answered the question with a range of responses. In total, 23 codes were identified from 155 open‐ended responses. These codes were then summarized and grouped further to create 7 distinct themes, which are specific patterns that were found in this dataset. Firstly, “concerns with CAIM categorization” included individuals who disagreed with the grouping of CAIM modalities in the survey and believed that some categories were too broad or too narrow. Secondly, “CAIM is effective if beneficial to the patient and integrated into practice” included individuals who believed that efficacy was of foremost concern, and that patient results matter most. Another category was “CAIM should be studied using evidence‐based methods”, which included responses that advocated for further research and for rigorous scientific evidence. The next category was “polarized opinions about CAIM” which included individuals who were either for or entirely against CAIM modalities being integrated into treatment. Finally, “physicians require CAIM education” encompassed responses that described the need for further education in various levels of institutions regarding CAIM. Coding and thematic analysis data are available at: https://osf.io/d45z6.

## 4 Discussion

The purpose of this study was to explore the perceptions of pain researchers and clinicians about CAIM. Participants found mind–body therapies to be the most promising CAIM category and the area that the clinician/researchers had practiced most frequently or recommended to patients. On the contrary, participants were more skeptical about biofield therapies and biologically based practices. Participants also agreed that conducting further research on mind–body therapies and manipulative and body‐based practices is the most valuable, and that clinicians should receive training on CAIM therapies via formal or supplementary education.

### 4.1 Comparative Literature

The findings from our survey align with previous literature examining researcher and clinician perceptions of CAIM. Across researchers and clinicians in the medical specialties of psychiatry, oncology, neurology, pediatrics, cardiology, and surgery, mind–body therapies were the CAIM modality perceived as the most promising, while biofield therapies were perceived as the least promising [26, 27, 28, 29, 30, 31].

In our study, mind–body therapies (e.g., yoga, meditation, biofeedback, tai chi) were considered the most promising with regards to prevention, treatment, and management of pain, and biofield therapies were perceived as the least promising. This is consistent with other studies conducted within the field of pain. One systematic review found that mind–body therapies were associated with moderate improvements in pain and small reductions in opioid dose among adults prescribed opioids for clinical pain. The study highlighted that meditation, hypnosis, suggestion, and cognitive behavioral therapy were particularly effective, and that these therapies may provide additional benefits such as reducing opioid craving and misuse, which is highly relevant in the context of the opioid crisis [32]. A systematic review of meta‐analyses reported that mind–body interventions produced small to moderate reductions in depressive symptoms among people with chronic pain, indicating consistent psychosocial benefits. Their systematic review also emphasized that current evidence is limited and further clinical trials are needed, particularly for patients with axial pain or comorbid major depressive disorder, to strengthen guidance for clinical practice [33]. Another systematic review and meta‐analysis demonstrated that mind–body therapies may be effective in improving cancer‐related pain, with their meta‐analysis showing a significant reduction in pain intensity across multiple settings. However, they also noted that most studies were of low quality, with high risk of bias, highlighting the urgent need for further high‐quality trials to confirm these findings [34]. In contrast, a scoping review found that biofield therapies, despite substantial research activity including hundreds of randomized and controlled trials, showed inconsistent results across various health conditions and patient populations. Their scoping review underscored that challenges such as inconsistent reporting, diverse interventions, and methodological limitations hinder the integration of biofield therapies into mainstream medical practice and limit confidence in their clinical efficacy [35].

In our study, like in other studies, participants expressed that conducting research on CAIM is valuable and that additional funding should be directed toward this area. Given that our sample primarily comprised those who are researchers or who were both researchers and clinicians, it is reasonable that they would emphasize the importance of further research. Participants also supported the inclusion of formal or supplementary CAIM training for clinicians, which is consistent with findings from studies also conducted among those in other medical specialties [26, 27, 28, 29, 30, 31]. In the present study, most respondents in our study were senior researchers and clinicians who likely completed their professional training at a time when CAIM therapies were less integrated into mainstream healthcare, which may explain their support for incorporating CAIM education into medical training programs. The thematic analysis further revealed that participants viewed the limited availability of high‐quality evidence as a major barrier to the acceptance of CAIM, reinforcing the need for continued rigorous research in this field.

With respect to future directions, research should investigate how clinician and researcher perceptions toward CAIM shape clinical decision‐making, influence research priorities, and inform policy development. Further studies could also focus on designing and evaluating targeted educational programs aimed at improving clinician knowledge, competence, and confidence in incorporating CAIM approaches into pain management.

## 5 Strengths and Limitations

This study provides a generalized understanding of researchers' and clinicians' perceptions of CAIM due to the large and international sample of individuals possessing varying opinions on the topic. The email addresses and names of participants were found using MeSH headings Medical Subject, as it is an effective way of categorizing articles [36]. To maximize the response rate, we sent out multiple reminders via email throughout the survey duration. We only sent emails to individuals who have published over the last seven years, thereby limiting the potential for inactive addresses and maximizing response.

Several potential biases should be considered when interpreting the findings of this study. First, because only corresponding authors of English‐language journal articles were sampled and the survey was administered in English, our respondent pool likely overrepresented individuals who are proficient in English and underrepresented non‐English‐speaking researchers. Additionally, corresponding authors tend to hold more senior academic or professional positions, which may have introduced a bias toward more experienced researchers whose perspectives could differ from those of early‐career scholars. Beyond these sampling biases, respondents' views may have been influenced by professional or financial interests. For example, clinicians who use or profit from CAIM therapies, or conversely, those who benefit from promoting conventional treatments or critiquing CAIM practices. Moreover, respondents' prior research experiences could have shaped their opinions; those with positive, negative, or null findings in their own CAIM studies may interpret the evidence base differently. Notably, 42% of respondents reported never conducting CAIM research, suggesting that a substantial portion of responses may have been informed by anecdotal rather than empirical understanding of evidence. Furthermore, most respondents were based in North America and Europe, resulting in limited representation from other world regions. This geographic concentration may have biased the findings toward perspectives more reflective of Western world research and clinical contexts, which could differ from those in regions where CAIM practices are more culturally integrated or differently regulated. Finally, because the sampling method was based on publication volume, areas of CAIM with greater research output likely yielded more respondents familiar with those topics, potentially leading to greater confidence in more widely studied modalities and less confidence in less‐researched ones. Collectively, these biases highlight important caveats to the interpretation of our findings and underscore the need for caution when generalizing the results to the broader CAIM research community. Our response rate is likely underestimated due to certain email addresses being inactive or invalid; additionally, some individuals were inevitably unable to respond for a range of reasons, such as being on vacation, out of office, retired, or having passed away. There were also many non‐responding participants who received the email but did not complete the survey, which is often an inherent limitation of collecting data utilizing a survey. Nonresponse bias could have occurred when the characteristics of the non‐responders vary from that of the responders [37]. There could have been a potential for recall bias as participants might have struggled with recounting certain information. The study was also susceptible to sampling bias as we selected subjects from a large population [38]. Nevertheless, the overall response rate of 2.6% represents a limitation that may affect the representativeness and generalizability of the study findings.

## 6 Conclusions

This study provides a comprehensive, international perspective on researchers' and clinicians' perceptions of CAIM for pain. Mind–body therapies and manipulative and body‐based practices were viewed as the most promising modalities, whereas biofield therapies elicited greater skepticism. Participants emphasized the value of conducting rigorous research and advocated for increased funding to strengthen the evidence base. Additionally, there was strong support for integrating CAIM education into formal and supplementary clinician training, highlighting the importance of equipping healthcare professionals with the knowledge and skills to counsel patients effectively. Despite limitations related to sampling, geographic representation, and potential biases, these findings underscore the need for continued research, evidence‐based implementation, and targeted education to inform safe, effective, and patient‐centered integration of CAIM into pain care.

## Author Contributions

**Jeremy Y. Ng:** designed and conceptualized the study, collected and analyzed data, drafted the manuscript, and gave final approval of the version to be published. **Dennis Anheyer:** assisted with the analysis of data, made critical revisions to the manuscript, and gave final approval of the version to be published. **Holger Cramer:** assisted with the analysis of data, made critical revisions to the manuscript, and gave final approval of the version to be published. All listed authors have contributed to the manuscript substantially and have agreed to the final submitted version.

## Funding

The authors have nothing to report.

## Ethics Statement

We sought and were granted ethics approval by the University Hospital Tubingen Research Ethics Board prior to beginning this project (REB Number: 389/2023BO2).

## Consent

This study did not involve patients. All participants consented to taking part in the survey prior to participating.

## Conflicts of Interest

The authors declare no conflicts of interest.

## Data Availability

The data that support the findings of this study are openly available in Open Science Framework at https://doi.org/10.17605/OSF.IO/DVECG.
